# Nigeria *Anopheles* Vector Database: An Overview of 100 Years' Research

**DOI:** 10.1371/journal.pone.0028347

**Published:** 2011-12-05

**Authors:** Patricia Nkem Okorie, F. Ellis McKenzie, Olusegun George Ademowo, Moses Bockarie, Louise Kelly-Hope

**Affiliations:** 1 Institute for Advanced Medical Research and Training, College of Medicine, University of Ibadan, Ibadan, Nigeria; 2 Fogarty International Center, National Institutes of Health, Bethesda, Maryland, United States of America; 3 Centre for Neglected Tropical Diseases, Liverpool School of Tropical Medicine, Liverpool, United Kingdom; Louisiana State University, United States of America

## Abstract

*Anopheles* mosquitoes are important vectors of malaria and lymphatic filariasis (LF), which are major public health diseases in Nigeria. Malaria is caused by infection with a protozoan parasite of the genus *Plasmodium* and LF by the parasitic worm *Wuchereria bancrofti*. Updating our knowledge of the *Anopheles* species is vital in planning and implementing evidence based vector control programs. To present a comprehensive report on the spatial distribution and composition of these vectors, all published data available were collated into a database. Details recorded for each source were the locality, latitude/longitude, time/period of study, species, abundance, sampling/collection methods, morphological and molecular species identification methods, insecticide resistance status, including evidence of the kdr allele, and *P. falciparum* sporozoite rate and *W. bancrofti* microfilaria prevalence. This collation resulted in a total of 110 publications, encompassing 484,747 *Anopheles* mosquitoes in 632 spatially unique descriptions at 142 georeferenced locations being identified across Nigeria from 1900 to 2010. Overall, the highest number of vector species reported included *An. gambiae* complex (65.2%), *An. funestus* complex (17.3%), *An. gambiae* s.s. (6.5%). *An. arabiensis* (5.0%) and *An. funestus* s.s. (2.5%), with the molecular forms *An. gambiae* M and S identified at 120 locations. A variety of sampling/collection and species identification methods were used with an increase in molecular techniques in recent decades. Insecticide resistance to pyrethroids and organochlorines was found in the main *Anopheles* species across 45 locations. Presence of *P. falciparum* and *W. bancrofti* varied between species with the highest sporozoite rates found in *An. gambiae* s.s, *An. funestus* s.s. and *An. moucheti*, and the highest microfilaria prevalence in *An. gambiae* s.l., *An. arabiensis*, and *An. gambiae* s.s. This comprehensive geo-referenced database provides an essential baseline on *Anopheles* vectors and will be an important resource for malaria and LF vector control programmes in Nigeria.

## Introduction

Malaria and lymphatic filariasis (LF) are the two most important vector borne parasitic diseases worldwide [1], [2]. In Africa these diseases are both primarily transmitted by *Anopheles* species. Nigeria has the largest burden of malaria and lymphatic filariasis in Africa – yet very little is known about the distribution of *Anopheles* mosquitoes that act as vectors for both diseases, how the species interact, overlap or differ across the country [1], [2]. Knowledge of the geographical distribution of the different species, their ecological parameters, role in transmission, and susceptibility to insecticide-based interventions is critical if malaria and LF are to be controlled and eliminated in the next decade [3].

The World Health Organization's (WHO) Position Statement on Integrated Vector Management to control malaria and lymphatic filariasis promotes integrated vector management (IVM) to improve the cost effectiveness of vector-control operations, and to strengthen the capacity of programmes, partnerships and intersectoral collaboration in their efforts to control vector-borne diseases [3]. There is overlapping geographical distribution of malaria and LF in large areas of Africa, and where *Anopheles* mosquitoes transmit both the malarial and lymphatic filariasis parasites, scaling up vector-control methods such of insecticide-treated mosquito nets (ITNs) and implementing indoor residual spraying (IRS) for malaria control can effectively reduce transmission of LF [3]–[5].

Malaria is caused by infection with a protozoan parasite of the genus *Plasmodium* and is endemic in 106 countries and responsible for about 225 million clinical cases and 781,000 deaths annually [1]. The short-term goal of WHO's Global Malaria Programme is to reduce the burden of malaria until it is no longer a public-health problem, while the long-term goal is to reduce the global incidence to zero by progressively eliminating the disease in endemic countries [3]. The two main components of this programme are vector control and appropriate case-management through diagnosis and treatment [3], [6]. Malaria vector control involves a two pronged approach: (1) use of ITNs and/or long lasting insecticide nets (LLINs); and (2) IRS with insecticides [3], [6]–[7].

International funding for malaria control has risen steeply in the past decade and has led to rapid scale-up of ITNs in Africa through support from various donors, including the Global Fund, World Bank, UNITAID, UNICEF, DfID, USAID and Canadian Red Cross, as well as funds from governments and other development agencies [8], [9]. While this increasing trend in vector control is encouraging, there is still a need to reach more households given that in recent years, an estimated 42% of households in Africa owned at least one ITN, and only 10% received IRS [1], [3]. By the end of 2009 a total of 19,300,000 ITNs and/or LLINs had been distributed in Nigeria and 330,000 people were protected by IRS but this was still significantly below the WHO target for 2010. Only 15% of the Nigerian population (100% at risk) owned an ITN [1]. Also critical is the need for monitoring and evaluation systems to assess the impact and efficacy of these interventions. Current vector control methods involving ITNs and IRS primarily use pyrethroid insecticides although bendiocarb and DDT (Dichloro-Diphenyl-Trichloroethane) are used in some areas [10]. This widespread use of a single class of insecticide could give rise to the development of insecticide resistance in the mosquito vectors and lead to a major public health problem given the limited availability of alternative insecticides [10]–[12]. In Nigeria the entire population (154,728,895) is at risk of malaria and in 2009 there were 4,295,686 confirmed cases, 658,732 inpatient malaria cases and 7,522 malaria attributed deaths [National/Nigeria Malaria report; 1]. The National Malaria Control Programme (NMCP) of Nigeria has various intervention policies and strategies. These include the following: distribution of ITNs or LLINs; IRS with insecticides; intermittent preventive treatment (IPT) during pregnancy; and malaria case management.

Lymphatic filariasis is caused by parasitic worms that are transmitted by mosquitoes. About 90% of infections are caused by *Wuchereria bancrofti*, while most of the remainder are caused by *Brugia malayi*. In Africa, the major vectors of *W. bancrofti* are mosquitoes of the genera *Anopheles* and *Culex* [2]. The Global Program to Eliminate Lymphatic Filariasis (GPELF) was launched in 2000 with the aim of (1) interrupting transmission; and (2) reducing morbidity and preventing disability [2], [3]. Interrupting transmission between mosquitoes and humans is possible through mass drug administration (MDA), using once-yearly treatment with a single dose of albendazole plus either ivermectin or diethylcarbamazine (DEC) for 4–6 years [2], [3].

The GPELF has scaled up rapidly and by the end of 2009, 52 out of 81 endemic countries were implementing MDA, and 2.7 billion treatments had been delivered to 695 million people [3]. Although significant progress has been made in the use of MDA for LF control, the role of vector control is an increasingly important issue for meeting the challenges of eliminating the disease [3], [13]. Vector control is recommended as a possible strategy for controlling LF in some countries where LF and *Loa loa* are co-endemic and where the burden of LF is heaviest e.g, Nigeria, Democratic Republic of the Congo, India, Indonesia and Bangladesh [3]. Reducing mosquito populations through the use of ITNs and other insecticide treated materials, as well as IRS, may help accelerate or sustain the interruption of transmission. In the year 2000, the WHO African Region launched the Programme for Elimination of Lymphatic Filariasis, and started MDA in four countries (Ghana, Nigeria, Togo and the United Republic of Tanzania). This programme has since expanded, and in 2009 a total of 19 African countries had started MDA implementation [2]. However MDA coverage in Nigeria is still less than 100% of the geographical area, which is of concern given that the country has the highest burden of the disease in Africa [2].

Several maps of malaria and LF vector spatial distributions in Africa have been produced, however, most are at continental or sub-regional scale with limited specific data available for further use by national programmes and researchers aiming to better understand the epidemiology of the diseases [14]–[19]. National vector spatial distribution maps and small databases have been developed for Ghana [20], Kenya [21], and Mali [22], [23]. However, to the best of our knowledge, no country has specifically developed a national database for all *Anopheles* vectors which includes extensive historical data and specific information on locations, methodologies, insecticide resistance and parasite prevalence available in the public domain.

In Nigeria, recent studies have identified mosquitoes of the *An. gambiae* (principally *An. gambiae* s.s. and *An. arabiensis*) and *Anopheles funestus* complexes as the main vectors of malaria and LF [18], [24]. *An. melas* is found in the coastal areas and is involved in malaria transmission [25]–[27]. Most entomological studies have focused on small area/district based collections except for a few studies [26], [28]–[31]. The most extensive data available are by Service [32] who described the distribution of 29 distinct *Anopheles* species across the country in the 1960s, and more recently by Awolola et al. [33] who developed a Malaria Entomological Profile for Nigeria.

The aim of this study was to compile a national database on the *Anopheles* vectors of malaria and LF, including related information on the location, time/period of study, species abundance, sampling and collection methods, morphological and molecular species identification methods, insecticide resistance status, including evidence of the kdr allele, and *P. falciparum* sporozoite rate and *W. bancrofti* microfilaria (mf) prevalence. This database will provide an essential baseline on *Anopheles* vectors and will be an important resource for malaria and LF vector control and elimination programmes in Nigeria.

## Methods

### Study area

Nigeria is a federal constitutional republic comprising thirty-six states and its Federal Capital Territory, Abuja. The states are grouped into six geopolitical zones (northwest, northeast, north central, southwest, southsouth and southeast) [34], [35]. The main latitude and longitude of Nigeria is 10° North and 8° East respectively [36]. Nigeria is approximately 923,768 sq. km and it is located in West Africa and shares borders with Benin in the west, Chad and Cameroon in the east, and Niger in the north [34]. Its coast in the south lies on the Gulf of Guinea on the Atlantic Ocean. There are two main seasons: the wet and the dry season. Most of the rainfall in Nigeria occurs between June and September. Nigeria is broadly grouped into two zones: forests and savanna [35], [37]. The following vegetation types are recognized in the country: the mangrove and fresh water swamps, the rain forest, the Guinea savanna, the Sudan savanna and the Sahel in a south-north transect. Between the rain forest and the Guinea savanna is a modified vegetation transition consisting of light deciduous forest and derived savanna [34].

### Data collection/collation

A systematic collation of primary empirical occurrence data for *Anopheles* mosquitoes in Nigeria, in published articles, was carried out to develop a comprehensive geo-referenced database of the distribution of *Anopheles* mosquitoes. The search was conducted using electronic searches in online bibliographic archives i.e. PubMed, SCOPUS and The Walter Reed Biosystematics Unit Culicidae Systematic Literature Database. Search terms, and combinations thereof, included Nigeria, *Anopheles*, mosquito, vectors, malaria, lymphatic filariasis. All articles with information on *Anopheles* were included. Many articles were identified through this method and many of the references were obtained from the references listed within articles, and then from the references within those articles and so on. Articles that could not be obtained online were sourced from three main libraries: College of Medicine Library, University of Ibadan; Liverpool School of Tropical Medicine Library, and the National Institute of Health Library. The references of articles obtained were also searched for additional sources of information.

For each article the following information was recorded: the locality, latitude and longitude, time/period of study, year project was initiated, species, number of specimens recorded, collection method (animal baits, human baits, indoor/outdoor resting collections, bednet trap, exit traps, human landing catches, and pyrethrum spray catches), stage of collection (adult or larval), morphological identification method (cytogenetic analysis of polytene chromosome, cross mating, morphology), molecular identification method, insecticide resistance status, insecticide tested, presence or absence of the kdr allele, *P. falciparum* sporozoite rate, *W. bancrofti* microfilaria prevalence and the reference. Only articles containing information related to the aim of this study were included in the final analysis.

The locations (i.e. mosquito collection sites) were geo-referenced using the latitude and longitude coordinates obtained by cross-checking the names with data from the GEOnet Names Server [36], Directory of Cities and Towns in the World [38] databases. Some site locations were also obtained from the research articles, as provided by the authors, while others were obtained from the WHO Malaria Entomological Profile for Nigeria [33] and other internet sources. Degree/minutes/seconds were converted into decimal degrees. It is acknowledged that there are limitations in using geographical coordinates obtained retrospectively; however, we have listed the main source of coordinates in the absence of such data. All the relevant information was entered into an Excel spreadsheet and data analysis was performed using SPSS (Version 15 for Windows, SPSS Inc., Chicago, IL). All data were mapped using the geographical information systems software ArcGIS 9.2 (ESRI, Redlands, CA). The overall species distributions and levels of insecticide resistance of the main *Anopheles* species were mapped and compared between two time periods i.e. before and after 2000. The total number of studies carried out was also compared between the six geopolitical zones and between time periods. All other data were tabulated or graphed to highlight differences in sampling, collection and identification methodologies, information related to insecticides and parasite prevalence. The mean, minimum and maximum sporozoite and microfilariae prevalence rate was calculated using SPSS software and was calculated according to species.

## Results

### Mosquito species and distribution maps

In total, 110 publications reporting on a total of 484,747 *Anopheles* mosquitoes from 632 spatially unique descriptions at 142 geo-referenced locations across Nigeria were identified between 1900 and 2010 (full database available in Table S1). Overall, the vector species most often reported included *An. gambiae* complex (65.2%), *An. funestus* complex (17.3%), *An. gambiae* s.s. (6.5%) *An. arabiensis* (5. 0%) and *An. funestus* s.s. (2.5%). Other species (4.5%) included *An. coustani*, *An. hancocki*, *An. leesoni*, *An. nili*, *An. melas*, *An. moucheti*, *An. rivulorum* and *An. wellcomei*.

For the time period 1900–1999, a total of 420 species-specific data points (n = 422,137 *Anopheles* mosquitoes) were recorded across 66 geo-referenced locations from 63 references, while for the time period 2000–2010, a total of 212 data points (n = 62,610 *Anopheles* mosquito) were recorded across 82 geo-referenced locations from 48 references. A higher proportion of data is recorded for the *An. gambiae* and *An. funestus* complexes in 1900–1999, while more data is recorded for *An. gambiae* s.s. and *An. funestus* s.s. in 2000–2010.

**Figure 1:**
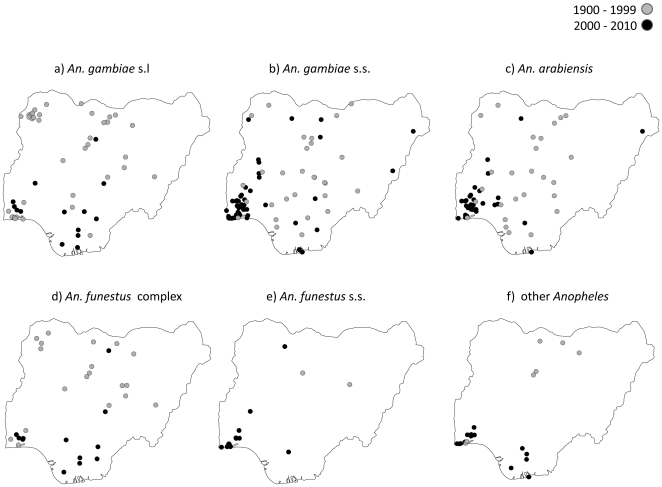
Distribution of *Anopheles* species a. *An. gambiae* s.l. b. *An. gambiae* s.s. c. *An. arabiensis* d. *An. funestus* complex e. *An. funestus* s.s. f. Other *Anopheles*.

**Figure 2:**
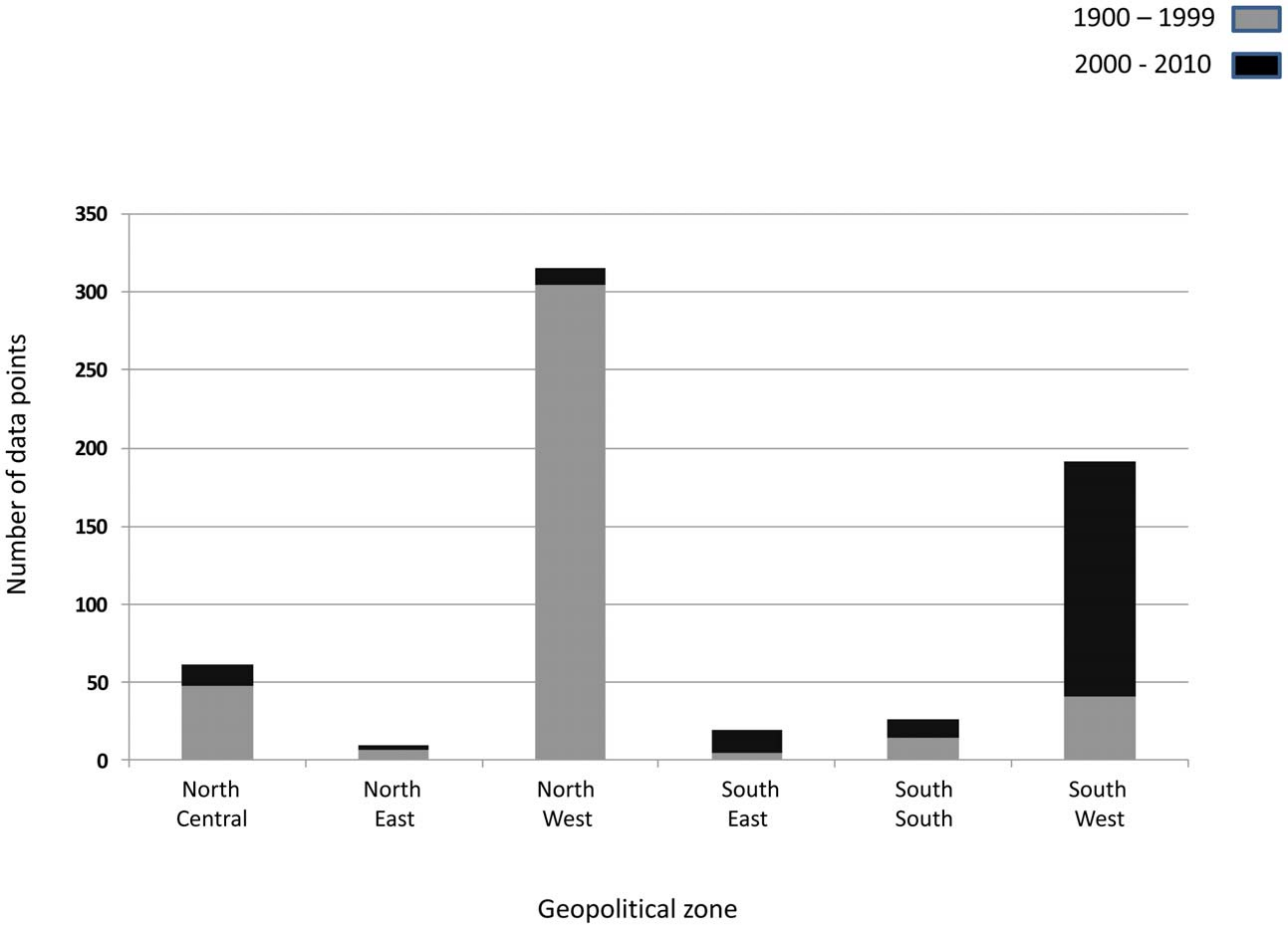
Number of data points across six geopolitical zones by time period.

The distribution of each species and locations across the two different time periods is summarised in Table 1 and Figure 1a–f. Of the total 632 data points recorded, 50% (316/632) and 30.5% (193/632) were from the northwest zone and southwest zone respectively (Figure 2). The proportion of studies that were carried out in the north central, south south, southeast and northeast zones of the country was 10.1%, 4.3%, 3.5% and 1.6% respectively. The majority of the studies in the northwest zone were carried out before, rather than after the year 2000: 72.6% (305/420) versus 5.2% (11/212). However, the reverse was the case in the southwest zone, where the majority of the studies was carried out after, rather than before the year 2000: 71.7% (152/212) versus 9.8% (41/420).

**Table 1:** The number and proportion of *Anopheles* species found in studies between 1900 and 2010.

	1900–1999		2000–2010		Total	
Species(n = data points)	N	%	N	%	N	%
*An. gambiae* s.l (181)	302677	71.7	13472	21.5	316149	65.2
*An. gambiae* s.s. (156)	8546	2.0	22760	36.4	31306	6.5
*An. arabiensis* (122)	19529	4.6	4634	7.4	24163	5.0
*An. funestus* complex (95)	79998	19.0	4064	6.5	84062	17.3
*An. funestus* s.s. (21)	2382	0.6	4946	1.7	7328	2.5
Other species (57)	9005	2.1	12734	20.3	21739	4.5
**Total (632)**	**422,137**	100.0	**62,610**	100.0	**484,747**	100.0

The maps in Figure 1a–f show the spatial and temporal differences of each main species, and highlight the focus in the southern regions of country in the past decade. Figure 1d–e also shows that very few studies have been carried out on the *An. funestus* complex and *An. funestus* s.s. The number of data points for all species by each year and time period are summarised in Table S2 and S3.

**Figure 3:**
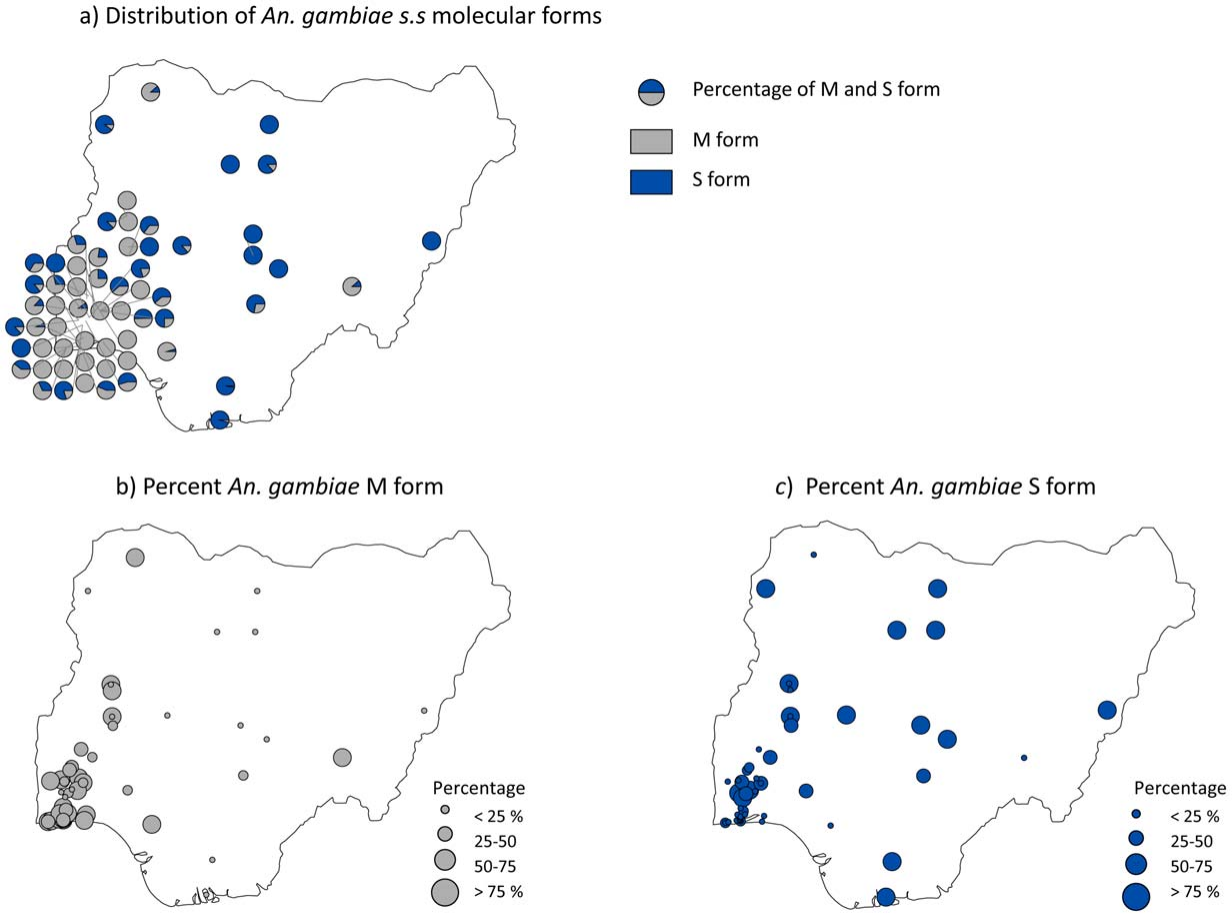
a. Distribution of *An. gambiae* s.s. molecular forms. b. Percent of M form c. Percent of S form.

The total number of *An. gambiae* s.s. molecular forms M and S form collected throughout the study period was 4784 and 5224 (Table 2) across 59 and 61 geo-referenced locations, respectively. For the time period 1900–1999, a total of 170 and 244 species specific data points of *An. gambiae* M form and S form were collected (Table S4), compared with the time period 2000–2010, when a total of 4614 and 4980 data points of *An. gambiae* M form and S form were collected, respectively (Table S5). Figure 3 a–c shows the distribution of the molecular forms and the percentage contribution of each molecular form.

**Table 2:** The number and proportion of *An. gambiae* s.s. molecular forms found in studies between 1900 and 2010.

	1900–1999		2000–2010		Total	
*An. gambiae* s.s(n = data points)	N	%	N	%	N	%
M form (61)	170	41.1	4614	48.1	4784	47.8
S form (59)	244	58.9	4980	51.9	5224	52.2
**Total (120)**	**414**	100.0	**9594**	100.0	10008	100.0

### Mosquito sampling and collection methods

Overall, the most used method of collecting *Anopheles* mosquitoes was via the adult stage. Chronologically (based on the year each project was initiated) adult collection represented 87.5% (7/8), 100% (3/3), 67.1% (47/70), 96.1% (221/230), 33.9% (41/121) and 36.2% (54/149) of collections made between 1900–1920, 1921–1940, 1941–1960, 1961–1980, 1981–2000 and 2001–2010 respectively. Larval collections represented 12.5% (1/8), 0% (0/3), 4.3% (3/70), 0.9% (2/230), 48.8% (59/121) and 34.2% (51/149) for the same time periods.

### 1900–1999

**Figure 4:**
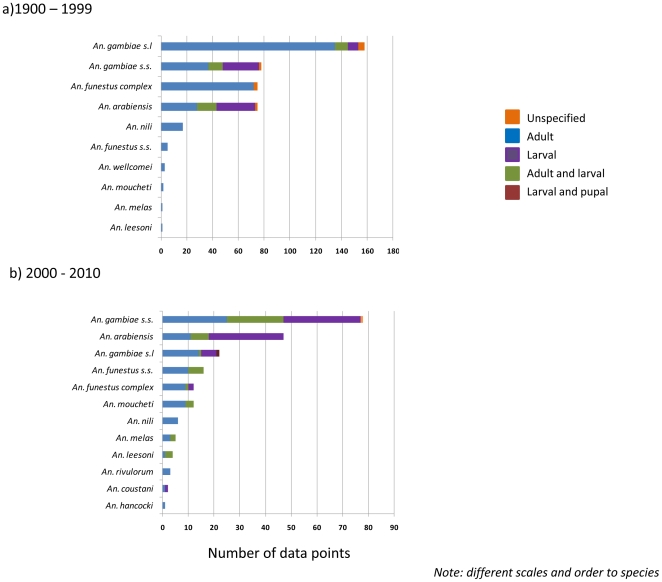
Summary of *Anopheles* species collection methods by time period a 1900–1999 b. 2000–2010.

The main methods of collecting *Anopheles* mosquitoes included adult collections (72.9%; 306/420) and larval collections (15.7%; 65/420), while a small proportion used larval and adult collections combined (8.6%; 36/420) or did not specify the specific methods (2.9%; 12/420) (Table 3). In general, the frequency of methods used was similar between the main species (Figure 4a).

**Table 3:** Summary of *Anopheles* species collection methods in studies between 1900 and 1999.

Species	Adult collection	Larval collection	Adult+larval collection	Unspecified	Total
*An. gambiae* s.l	135	8	10	5	158
*An. gambiae* s.s.	37	28	11	2	78
*An. arabiensis*	28	30	15	2	75
*An. funestus* complex	77	0	0	3	80
*An. funestus* s.s.	5	0	0	0	5
*An. leesoni*	1	0	0	0	1
*An. melas*	1	0	0	0	1
*An. moucheti*	2	0	0	0	2
*An. nili*	17	0	0	0	17
*An. wellcomei*	3	0	0	0	3
**Total**(n = data points)	**304**	**65**	**36**	**12**	**420**

Adult collections were made primarily from pyrethrum spray catches (PSC) (17.6%), human landing catches (HLC) (12.9%), indoor resting (IR) collections (8.6%), exit trap collections (ETC) (6.9%) and animal baits (1.7%). Some collections were made using a combination of methods e.g. net catch/human bait, pyrethrum spray catch/exit trap, human landing catch/pyrethrum spray catch.

Larval collections were made from potential breeding sites, which included gutters, abandoned road sides, standing waters, vehicle tracks, tires, shallow wells, ponds, swamps, drains, rivers, small streams, irrigation ditches, hoof prints, domestic containers and empty cans (Table S1). Some collections were made using a combination of larval and adult collection methods, e.g. pyrethrum spray catches/larval, indoor resting collections/larval, human landing catches/exit trap and indoor resting/human landing catch/pyrethrum spray catches/larval collections.

### 2000–2010

The main methods of collecting *Anopheles* mosquitoes included adult collections (45.3%; 96/212), larval collections (32.5%; 69/212), and larval and adult collections combined (21.2%; 45/212), while a small proportion used larval and pupal collections (0.5%; 1/212), or did not specify the methods (0.5%; 1/212), as shown in Table 4. Compared with data collection method in the 1900–1999 time period, overall there was a decrease in adult collections and an increase in the number of larval collections (Figure 4b).

**Table 4:** Summary of *Anopheles* species collection methods in studies between 2000 and 2010.

Species	Adult collection	Larval collection	Larval+pupal collections	Adult+larval collection	Unspecified	Total
*An. gambiae* s.l	15	6	1	1	0	23
*An. gambiae* s.s.	25	30	0	22	1	78
*An. arabiensis*	11	29	0	7	0	47
*An. funestus* complex	11	3	0	1	0	15
*An. funestus* s.s.	10	0	0	6	0	16
*An. leesoni*	1	0	0	3	0	4
*An. melas*	3	0	0	2	0	5
*An. moucheti*	9	0	0	3	0	12
*An. nili*	6	0	0	0	0	6
*An. rivulorum*	3	0	0	0	0	3
*An. coustani*	1	1	0	0	0	2
*An. hancocki*	1	0	0	0	0	1
**Total**(n = data points)	**96**	**69**	**1**	**45**	**1**	**212**

Adult collections were made primarily from human landing catches (18.4%), indoor resting collections (9.0%), CDC light traps (5.2%) and pyrethrum spray catches (5.2%). Some methods were also used in combination: human landing collections/pyrethrum spray catches/indoor resting collections, indoor resting/human landing catch.

Similar to the 1900–1999 time period, larval collections were made from a variety of potential breeding sites (Table S1), and some studies used a combination of adult and larval methods, e.g. human landing catch/larval/pyrethrum spray catches, larval/indoor resting, human landing catch/indoor resting/larval, larval/outdoor baited scarecrow, and larval/pupal collections.

### Species identification methods

Chronologically (based on the year each project was initiated), the method of species identification was not specified in studies initiated between 1900–1960 (n = 81). For 1961–1980 period, cytogenetic analysis of polytene chromosome represented 28.7% (66/230) of identification method employed. Morphological identification and use of PCR was the most used method of identification in studies carried out between 1981–2000 and 2001–2010 representing 60.3% (73/281) and 38.3% (57/149) respectively.

### 1900–1999

**Figure 5:**
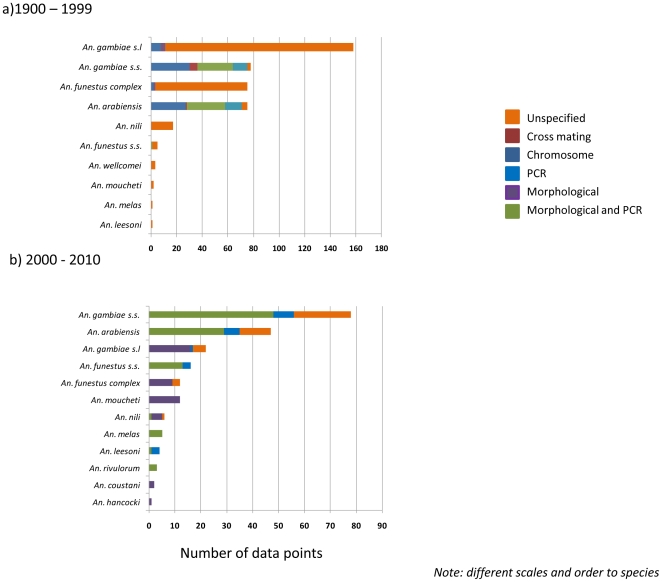
Summary of *Anopheles* species identification methods by time period a 1900–1999 b. 2000–2010.

The methods of species identification were based on cytogenetic analysis of polytene chromosome (16.0%; 67/420), morphology and PCR (14.0%; 59/420), PCR (6.0%; 25/420), cross mating techniques (1.9%; 8/420) and morphology alone (0.7%; 3/420) (Figure 5a). The method of species identification was not specified in studies initiated between 1900 and 1963 representing 61.4% (258/420) of sites. The earliest study that identified species based on cytogenetic analysis of chromosomes was in a study initiated in 1969 [39]; cross mating techniques was first used in a study initiated in 1963 [40], morphology was first specified in a study initiated in 1965 [41] while use of PCR was first mentioned in a study initiated in 1997 [29], [30].

The morphological keys used included those described in [42]–[45]. The molecular methods - polymerase chain reaction (PCR) assays for the vector species complex and molecular forms - were those of [46] for *An. gambiae* complex; [47] for distinguishing the molecular forms of *An. gambiae* s.s. and [48] for *An. funestus* complex.

For *An. gambiae* s.l. and the *An. funestus* complex, the species identification method was not specified for 93.0% (147/158) and 95.0% (76/80) of sites, respectively. The PCR method was not used at any site for the *An. gambiae* complex or other species, and only once for the *An. funestus* complex. In contrast, for *An. gambiae* s.s. and *An. arabiensis* the PCR method of [46] was used for species identification and the method of [47] for *An. gambiae* s.s molecular form identification. For the *An. gambiae* s.s. data, a total of 10 (n = 78) collection sites had information on both the M form and the S form.

### 2000–2010

The methods of species identification were based on morphology and PCR (47.6%; 101/212), morphology alone (21.7%; 46/212), and PCR alone (9.9%; 21/212) (Figure 5b). The method of species identification was not specified in 20.8% (44/212) of sites. The morphological keys used included [43]–[45], [49]–[58]. The molecular PCR methods for the identification of vector species complex and molecular forms were those of [46]–[48], [59]–[61] (Table S1).

For *An. gambiae* s.l. and *An. funestus* complex, morphological methods were used at 69.6% (16/23) and 73.3% (11/15) of sites, and not specified for 26.1 (6/23) and 20% (3/15) of sites respectively (Figure 5b). For *An. gambiae* s.s., *An. arabiensis* and *An. funestus* s.s., a combination of PCR and morphological identification was primarily used, while for other species morphological methods were primarily used (*An. coustani*, *An. leesoni*, *An. melas*, *An. moucheti*, *An. nili*, *An. hancocki* and *An. rivulorum*).

### Insecticide resistance

**Figure 6:**
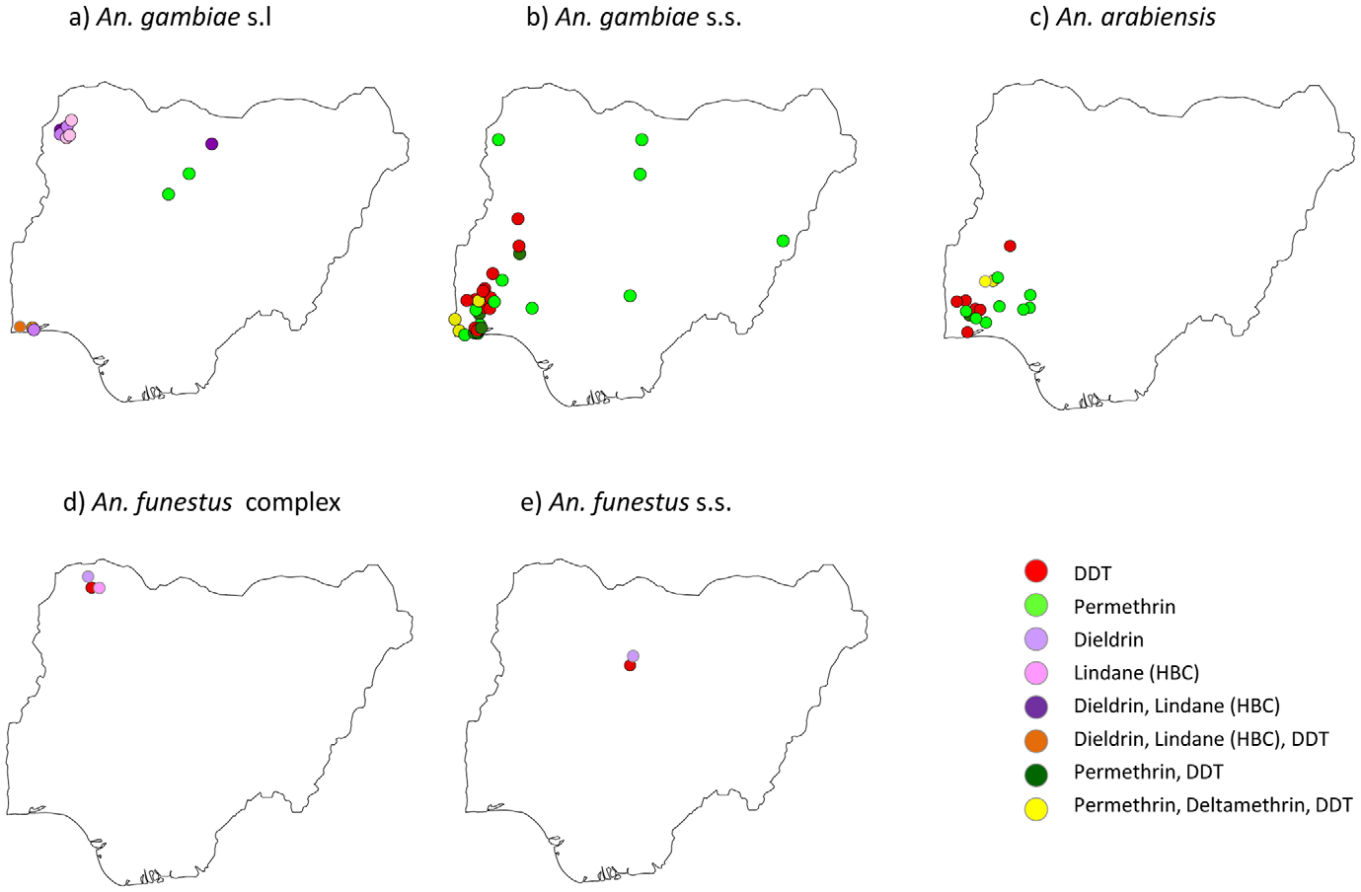
Distribution of insecticide resistance a. *An. gambiae* s.l. b. *An. gambiae* s.s. c. *An. arabiensis* d. *An. funestus* complex e. *An. funestus* s.s.

Insecticide resistance to pyrethroids and organochlorines was recorded in *An. gambiae* s.l., *An. gambiae* s.s., *An. arabiensis*, *An. funestus* complex and *An. funestus* s.s. across 45 geo-referenced locations (Table 5). The specific locations where resistance was found for each species, is available in Table S6 and highlighted in Figures 6a–g. The species most tested for insecticide resistance were *An. gambiae* s.s. 47.1% (33/70), *An. arabiensis* 25.7% (18/70) and *An. gambiae* s.l. 18.6% (13/70).

**Table 5:** Insecticides resistance recorded in *Anopheles* species.

Insecticide	*An. gambiae* s.l	*An. gambiae* s.s.	*An. arabiensis*	*An. funestus* complex	*An. funestus* s.s.	Total
BHC	3	0	0	1	0	4
DDT	1	12	6	2	1	22
Deltamethrin, Permethrin and DDT	0	4	0	0	0	4
Dieldrin	4	0	0	1	1	6
Dieldrin and BHC	3	0	0	0	0	3
Dieldrin, Lindane, DDT	1	0	0	0	0	1
Permethrin	1	12	10	0	0	23
Permethrin and DDT	0	5	2	0	0	7
**Total** (n = data points)	**13**	**33**	**18**	**3**	**2**	**70**

**Figure 7:**
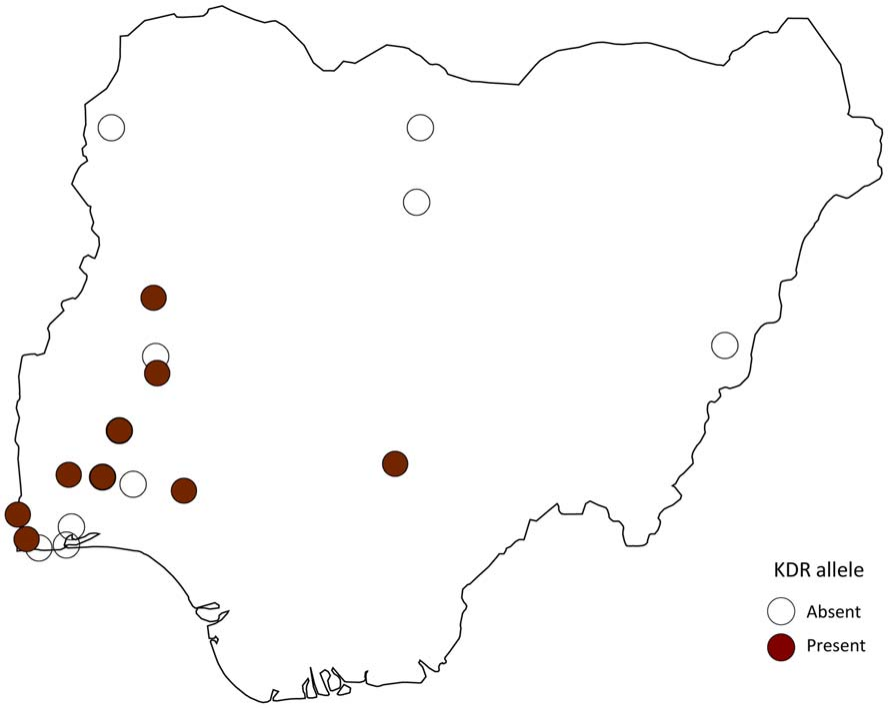
Distribution of kdr alleles.

Data were recorded for individual insecticides as well as a combination of insecticides. These were predominantly permethrin (32.9%; 23/70), DDT (31.4%; 22/70), and permethrin and DDT (10.0%; 70), as well as dieldrin (8.6%; 6/70), deltamethrin and permethrin and DDT (5.7%; 4/70), lindane (BHC) (5.7%; 4/70), dieldrin and lindane (BHC) (4.3%; 3/70), and dieldrin and lindane (BHC) and DDT (1.4%; 1/70) (Table 5). For *An. gambiae* s.s., resistance to DDT and permethrin was mainly reported, and the kdr allele was tested 26 times and found to be present in ten locations predominantly in the southwestern region of the country (Figure 7). For *An. arabiensis*, resistance to permethrin, and for *An. gambiae* s.l resistance to lindane (BHC), and dieldrin were mainly reported (Table S1).

### *Plasmodium falciparum* sporozoite rate

**Figure 8:**
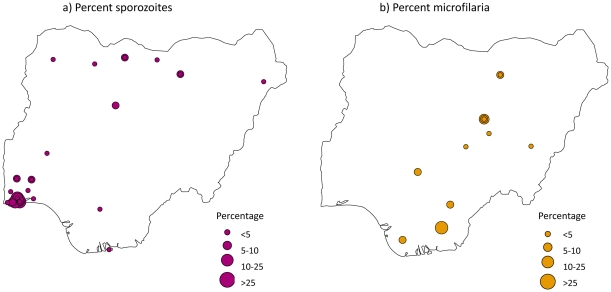
Distribution of a. sporozoite rates and b. LF microfilariae.

In total, *P. falciparum* sporozoite rates were recorded at 106 data points and geo-referenced locations from 20 references, and most of these were carried out in the last decade. Overall, the sporozoite rate varied by *Anopheles* species (Table 6) and location, with most data recorded in the southwestern region of the country (Figure 8a). Approximately 35% (30/86) of data points were recorded in two of the studies [62], [26].

**Table 6:** Mean sporozoite rate recorded for the various *Anopheles* species (data point = 106).

Species(n = data points)	Mean sporozoite rate (%)	Min sporozoite rate (%)	Max sporozoite rate (%)
*An. gambiae* s.l (32)	2.81	0.4	10
*An. gambiae* s.s (20)	19.4	0	91
*An. arabiensis* (11)	2.34	0	6.1
*An. funestus* complex (10)	1.99	0	5.35
*An. funestus* s.s (13)	11.79	0	50
*An. leesoni* (1)	0	0	0
*An. melas* (5)	2.57	0.4	6.5
*An. moucheti* (7)	5.22	0	21.9
*An. nili* (2)	0	0	0
*An. rivulorum* (3)	0	0	0
*An hancocki* (1)	0	0	0
*An. wellcomei* (1)	0	0	0

The mean sporozoite rate in *An. gambiae* s.l. was 4.3% and ranged from 0.4% (Sokoto [63]) to 10% (Lagos [64]). For *An. gambiae* s.s. the mean rate was 19.4%, and ranged from 0 (Bama [65]) to 91% (Alimosho [62]). For *An. arabiensis* the mean rate was 2.3% and ranged from 0 (Amuwo-Odofin [62] and Lemu surburb of Lagos [25]) to 6.1% (Kaduna area [39]). For the *An. funestus* complex, the mean rate was 2.1% and ranged from 0 (Sokoto [63]) to 5.35% (Kaduna area [66]). For *An. funestus* s.s. the mean rate was 11.8% and ranged from 0 (Agege) to 50% (Amuwo-Odofin) [62]. For *An. melas* was 2.57% and ranged from 0.4 (Bonny [26]) to 6.5% (Moba [27]). For *An. moucheti* the mean rate was 5.2%, and ranged from 0 (Remo north and Odogbolu LGA [67]) to 21.9% (Mushin [62]). For other species, no sporozoites were recorded for *An. leesoni*, *An. nili*, *An. rivulorum*, *An. hancocki* and *An. wellcomei* (Table 6).

### Lymphatic filariasis

The prevalence of LF microfilariae (mf) in *Anopheles* species was recorded for 21 data points, across ten geo-referenced locations and eight references (Table 7 and Figure 8b). The species found to be infected with mf included *An. gambiae* s.l., *An. gambiae* s.s., *An. arabiensis* and *An. funestus* complex. The mean mf prevalence in *An. gambiae* s.l was 5.64% and ranged from 0 (Seri [68] and Ibadan [69]) to 21.7% (Igwun Basin [70]). For *An. gambiae* s.s. and *An. arabiensis* mf was found only in one site and was 9.2% (Jos Plateau) and 11.1% (Jos Plateau) respectively [24]. For *An. funestus* complex the mean mf prevalence was 3.15% and ranged from 0% (Ibadan [69]) to 6.6% (Amassoma [71]). The data from two articles [68], [72] had to be excluded from the analysis because the general data on mf prevalence were combined from various sites and/or species. The infection rate of mosquitoes containing *W. bancrofti* was reported to be 1.0% for *An. funestus* complex and 3.55% for *An. gambiae* s.l but the particular site was not specified [68]. Also [71] gave the prevalence of mf to be 4.9% and 4.7% in the untreated and ivermectin treated village respectively, but the percentage contribution of each species was not given. These two papers both presented results from studies carried out in Plateau and Nassarawa states (Table S1).

**Table 7:** Prevalence of *W. bancrofti* microfilariae in various *Anopheles* species.

Species (data points)	Mean mf prevalence (%)	Min mf prevalence (%)	Max mf prevalence (%)
*An. gambiae* s.l (10)	5.64	0	21.7
*An. gambiae* s.s (1)	9.2	9.2	9.2
*An. arabiensis* (1)	11.1	11.1	11.1
*An. funestus* complex (9)	3.15	0	6.6

## Discussion

This collation of data from 110 publications has produced one of the most comprehensive geo-referenced databases on *Anopheles* vectors of malaria and filariasis available for any country in the world. Importantly, it has been carried out for Nigeria, which has the largest burden of both diseases in Africa, and is a priority country for international donors (the Global Fund, World Bank, UNITAID, UNICEF, DFID, USAID and Canadian Red Cross) supporting large-scale control programmes [9]. The data span more than 100 years and show changes in the geographical focus of research sites, field and laboratory methodologies, and they highlight critical gaps in our knowledge on *Anopheles* species distributions, transmission dynamics, species and parasite interactions, level and impact of insecticide resistance.

The results reported by the various articles show that the most abundant vectors are the *An. gambiae* complex, *An. funestus* complex, *An. gambiae* s.s., *An. arabiensis* and *An funestus* s.s., with these vectors existing in sympatry in most locations. The earliest study found was the expedition by the Liverpool School of Tropical Medicine [73]. In the earlier reports *An. gambiae* s.l. was referred to as “*A. costalis*” [74], [75]. In the early days (and early reports) the term “*An. gambiae*” encompassed the species complex [40], [63], [66], [76]–[91]. In subsequent reports *An. gambiae s.s.* was referred to as “species A” [92], [93] and *An. arabiensis* was referred to as “species B” [92], [93].

The total number of mosquitoes collected is under represented as the numbers of mosquitoes collected were not available in some studies. As the species abundance was not specified in some studies, the totals for each species was carried out independently and did not interfere with any of the calculations reported here. In general, there is more information provided on the *An. gambiae* s.s. and *An. funestus* s.s. collected in studies initiated in the 2000s than in those from earlier years. Also, more information was available on *Anopheles gambiae* s.l., which represented over 60% of the overall species composition compared to about 19.8% for *An. funestus* (17.5% of the studies were on *An. funestus* complex while 2.3% were on *An. funestus* s.s.). Clearly, research on the composition and distribution of the *An. funestus* group is important, as it is an important vector contributing to the transmission of both LF and malaria in Nigeria [26], [68], [93], [94]. One main reason for the lack of research on *An. funestus* may be that this vector is refractory to colonization, and very difficult to find in the field, and its larvae are very difficult to find at low densities due to their tendency to stay submerged for long periods [43]. However, new techniques for egg laying and colonising are being developed, which will facilitate research opportunities in the future [95].

Of particular interest is how the molecular forms of *An. gambiae* s.s. varied across the country. In most studies the *An. gambiae* M and S molecular forms were found in sympatry, although the ratio varied from location to location [30], [31], [96]–[101]. At the 120 sites where M and/or S forms were reported, pure populations of the S form were recorded at 7 sites, all located in the northern region (Guinea and Sudan savanna ecological zones) [30], [31], [102], [103]. Pure populations of the M form were reported at 17 sites, 14 of them located in the southern region (mangrove, forest, and transitional ecological zones) [27], [30], [101], [104], [105]. The other 3 sites with pure populations of the M form were from the north central (Guinea savanna) part of the country [101]. Geographical and ecological differences in the chromosomal and molecular forms of *An. gambiae* s.s. and their potential role in disease transmission have been examined in regions of West Africa [17], [20], [106] with the *An. gambiae* S form broadly associated with malaria distributions, and the *An. gambiae* M form with LF distributions. However, the role of these two sibling species in the transmission of malaria and/or LF and how they interact is largely unknown.

The series of mosquito vector distribution maps produced in this study highlight that studies in the 20th Century were carried out predominately in the northern region of the country, while studies carried out more recently have focussed mainly in the southern region. The reason for this shift may be related to limited infrastructure and an overall lack of trained staff, vector ecologists and medical entomologists, which are common problems across Africa [6], [7], [107], [108]. It may also be due to the limited accessibility of remote locations, as the majority of studies were carried out in proximity to cities and towns in two main zones, northwest and southwest, leaving large gaps in the eastern regions of the country. Furthermore, very few studies were carried out in the same locations in different years or seasons: this lack of systematic sampling/collections restricts analysis and comparisons on multiple fronts, for instance of changes in species composition and abundance over time. The data collated here can serve as a baseline for future work that includes such same-site collections and allows for comparisons within and between populations. Currently, this is particularly important given the widespread vector control interventions taking place across the country [9], [35].

There was great variability in the reporting of the mosquito sampling and collection methods used over time. For example, between 1900 and 1999, PSC, HLC, IR and ETC were the methods most frequently used either singly or in combination for adult mosquito collections, whereas in studies carried out between 2000 and 2010, the CDC light trap was mainly used; there was no record of ETC use during this period. Surprisingly, the expensive and labour intensive method of the HLC [109] was used consistently throughout the study period from 1900 to 2010, which is probably due to a lack of a suitable substitute for this important metric. However, recent tent traps have been tested and calibrated to the HLC and their use as an alternative tool is being trialled in different locations across Africa [109], [110]. The variability in the use of different sampling and collection methods calls for simpler and more standardized methods; the value of this has been previously emphasized [111]–[113].

Similarly, there were changes in species identification methods over time. Earlier studies used more cross mating techniques [40], [114], morphological [28], [41], [115], [116] and cytogenetic methods[39], [93], [117]–[122] for species identification, however, by the 2000s cross mating techniques and cytogenetic methods were nonexistent. Most of the missing data were from earlier studies when there were few options for species identification, For example, most of the missing data were from articles published between 1900 and 1963, when cross mating and morphological methods were predominately used. However it was interesting to note that taxonomic keys [49]–[52] were used for species identification in a study carried out between 2002 and 2003 [123]. In recent decades the emphasis has shifted to molecular techniques including PCR assays that target specific regions of repeat gene families, such as the ribosomal RNA (rRNA) gene family. A diagnostic assay for the identification of the *An. gambiae* complex based on IGS and ITS sequence differences was developed and applied routinely [46]. Similar techniques were also developed for *An. funestus* s.l. [48], and *An. moucheti* s.l. [61], [124], along with more advancements allowing the simultaneous identification the *An. gambiae* complex species and *An gambiae* s.s. M and S molecular forms [46], [47], [59].

In contrast, there were no major changes in the methods used for the detection of insecticide resistance in Nigeria. In most of the studies, bioassays and molecular assays to detect resistance alleles (specifically the kdr mutation) were used. Bioassays are the best indicators of the presence of resistance in a field population and are widely used across Africa [7], [11], [125], [126]. However, molecular and biochemical techniques are important to verify bioassay results of resistance in wild populations and gain an understanding of the underlying mechanisms of resistance [11], [12], [127]. In Nigeria only one study used bioassay and microarray analysis [105], and another used bioassays, molecular assays to detect resistance alleles, and biochemical analysis and microarray analysis to characterize pyrethroid resistance mechanism in colony breed *An. gambiae* s.s. [128]. The reason for the heavy reliance on bioassays and molecular assays for resistance monitoring is probably because the biochemical assays and microarray analysis require qualified personnel and specialized and costly equipment [10].

Insecticide resistance in the *An. gambiae* complex, *An. gambiae* s.s., *An.arabiensis*, *An. funestus* complex and *An. funestus* s.s. to various insecticides (DDT, permethrin, dieldrin, deltamethrin and lindane (BHC)) was widespread across the southwestern region of the country. Resistance was most frequently reported in *An. gambiae* s.s. to permethrin and DDT, which is of major concern given that this vector was found to have among the highest malaria sporozoite and LF microfilaria rates in Nigeria, permethrin is currently the only class of insecticide used for ITNs, and permethrin and DDT are widely used for IRS [10]. Vector control can be hampered by the occurrence of insecticide resistance and as such monitoring of insecticide resistance regularly across a wide geographical area is critical [7], [11], [12], [129]–[132]. The findings also highlight the importance of a consensus standardization of the number and location of surveillance sites and the frequency with which resistance monitoring should occur [11]. To date very little has been done in the northwestern region of Nigeria, especially, in the area where The Garki Project was carried out in the 1960–70s [93]. This is disappointing given that it was one of the largest and most comprehensive control programmes to be carried out in Africa at the time, and vector and resistance monitoring may have provided some invaluable insights into the long term effects of large scale vector control programmes.

In general, there were few studies that focussed on malaria and LF parasites. The sporozoite rates recorded emphasize the role of *An. gambiae* s.s. and *An. funestus* s.s. as very efficient malaria vectors. In areas of the southern part of the country when the two species were studied in sympatry, *An. gambiae* s.s. had higher sporozoite rates than *An. funestus* s.s. At Bungudu-Gusau in the northern part of the country, however, *An. funestus* s.s. had a sporozoite rate of 2.3% compared to 0.46% recorded in *An. gambiae* s.s. [26]. The sporozoite rates recorded for *An. arabiensis* were also lower than those for *An. gambiae* s.s. in areas where they were studied at the same time [26], [67], [133]. These findings call for a widespread systematic sampling across the country. For LF, the results show that *An. gambiae* s.l., *An. gambiae* s.s., *An. arabiensis* and *An. funestus* complex are important vectors. Of the eight studies [24], [68]–[71], [73], [75], [134] carried out to determine the prevalence of LF only one study [75] reported co-infection of *Plasmodium* parasites and LF in the mosquitoes that were trapped. Annett et al [73] confirmed the role played by *Anopheles* mosquitoes as malaria vectors but it was not stated whether co-infection of malaria parasites and LF was studied. The few records found make it difficult for comparisons to be made between studies, locations and species. This becomes alarming as Nigeria bears the greatest potential burden of LF in Africa, with 80 million people (19% of the total population) at risk [135].

As this study has restated that LF and malaria are transmitted by the same vectors in Nigeria, both diseases can be jointly controlled since the two diseases share a large proportion of their target population, and the national programmes have similar goals and strategies [3], [136]. The result from all the articles used in the database [24]–[31],[33],[40],[41],[61]–[94],[96]–[105],[114]–[124],[133]–[134],[137]–[177] emphasizes the need for a detailed understanding of the distribution, species composition, behaviour and insecticide susceptibility levels of local vectors in order to successfully control the diseases. It reveals the areas where a dearth of data exists and emphasizes the importance of collecting data systematically, so that the impact of the interventions can be measured. These considerations are important in the light of the goal of the WHO's Global Malaria Programme and the WHO's Global Programme to Eliminate Lymphatic Filariasis to eliminate the diseases as a public health problem [3]. The findings in this research shows that better linkages and partnerships between entomologists, parasitologists, national control programmes, international donors and other stakeholders in the country is needed, in order to carry out meaningful research and control on the vectors and their disease transmission across the country.

## Supporting Information

Table S1 Nigeria *Anopheles* vector database.
(XLSX)

Table S2 The number of data points by each species and each year from 1900–1999.
(XLSX)

Table S3 The number of data points by each species and each year from 2000–2010.
(XLSX)

Table S4 Number of specimen of *Anopeles gambiae* s.s. M form and S form from 1900–1999.
(XLSX)

Table S5 Number of specimen of *Anopeles gambiae* s.s. M form and S form from 2000–2010.
(XLSX)

Table S6 Localities and species recording resistance and the frequency.
(XLSX)
